# Necrology

**Published:** 1903-01

**Authors:** George H. Hart, T. E. Munce, Oscar Nelson, Harry E. States

**Affiliations:** Committee; Committee; Committee; President of the Society


### NECROLOGY.

The subject of this sketch, Mr. Frank Briard, was born July, 1878, at St. Heliers, Jersey, England. He was raised on his father's farm and educated in the schools of that country. In 1889 he entered St. James' Collegiate School, in Jersey, and continued until 1893, and at various times won awards of prizes in his class in many branches, especially in French translations. He was well versed in the care of cattle and the breeding of Jerseys. About five years ago he came to this country, and was employed for some time as herdsman on the celebrated Thorndale Dairy Farm, at Thorndale, Pa. Before entering the Veterinary Department of the University of Pennsylvania he took a business course at Storrs' College, at Storrs, Conn. During his period at the veterinary department he won the prize of his class for the best average in examinations of comparative anatomy, which course covers two years' study and dissection. He stood second for the highest general average in all branches for the two years completed June 1, 1902. Early in October he was taken with a severe attack of articular rheumatism followed by endocarditis. After ten weeks of illness at the University Hospital he died, January 3, 1903. His funeral was held and temporary interment made at Fernwood Cemetery, January 5th. Rev. Herbert Roche, of the Church of the Transfiguration, officiating, and about one hundred of his fellow students, professors, and friends attending the services. The following resolutions were read and adopted at the last regular meeting of the Veterinary Medical Society of the University of Pennsylvania, of which he was an active member and ex-librarian:

*Resolutions.*

Whereas, We, the assembled members of the Veterinary Medical Society of the University of Pennsylvania, wish to express our grief for the loss of our beloved fellow member and ex-librarian, Mr. Frank Briard.

WHEREAS, It has pleased Almighty God to have removed from us a most valued and earnest member of the society,

*Resolved,* That while we bow in submission to Heaven's decree, we feel deeply the loss of our friend, whose record as a student was beyond reproach, and as a friend and companion ever true.

*Resolved,* That we one and all sorrow for the suffering of our brother member, and that we tender our heartfelt sympathies to the bereaved family in their trying hour of affliction.

*Resolved,* That these resolutions be spread on the minutes of this society and that a copy be sent to the family in England, with whom we deeply sympathize.

GEORGE H. HART,
T. E. MUNCE,
OSCAR NELSON,
Committee.

HARRY E. STATES,
President of the Society.

